# Management of Thoracic and Cardiac Trauma: A Case Series and Literature Review

**DOI:** 10.7759/cureus.26465

**Published:** 2022-06-30

**Authors:** Akshay Kumar, Nimisha Shiwalkar, Sameer Bhate, Suresh Keshavamurthy

**Affiliations:** 1 Cardiothoracic Surgery, Lokmanya Tilak Municipal Medical College and General Hospital, Mumbai, IND; 2 Anesthesiology and Critical Care, University of Texas at Houston, Houston, USA; 3 Cardiothoracic Surgery, Ruby Hall Hospital, Pune, IND; 4 Surgery/Cardiothoracic, University of Kentucky, Lexington, Kentucky, USA

**Keywords:** cardiac troponin-t, echocardiography, focused assessment with-sonography in trauma, penetrating cardiac trauma, blunt cardiac trauma

## Abstract

Patients with cardiac and thoracic trauma remain one of the most difficult presentations to diagnose and treat in an emergency room setting. Here, we present our series of four cases of cardiac and thoracic trauma with varied presentations, including lung, vascular, and diaphragmatic injuries that were managed successfully. We further review the manifestations of cardiac trauma, including cardiac contusions, cardiac rupture, pericardial injury, and valvular injuries; thoracic trauma, including lung and diaphragmatic injury. The sheer complexity of the anatomical structures within the thorax makes it of the utmost importance to timely and appropriately manage them.

## Introduction

Although trauma to the heart was first mentioned in the year 3000 BC in medical literature, it remains one of the most complex and difficult conditions to treat. Furthermore, it was not until September 1896 that the first successful cardiac repair for trauma was performed by Dr. Ludwig Rehn in Frankfurt, Germany. With the development of cardiopulmonary bypass by Dr. John H. Gibbon in 1953, repair of various types of heart complications became possible and ushered in the modern era of treating cardiac trauma. In the present era, one of the most common sources of cardiac trauma is motor vehicle accidents (MVAs), which can often lead to hemorrhage and death [1]. MVAs also account for almost 81% of the total number of blunt cardiac trauma cases [1]. Approximately 10-25% of all mortalities due to trauma, MVAs included, are a result of cardiac and aortic injuries [2]. According to the World Health Organization (WHO), almost 1.3 million deaths were due to road traffic accidents in 2015, with 76% of victims being male [3]. The WHO also predicts that by the year 2030, road accidents will become the fifth leading cause of mortality [3].

Inconsequently, penetrating cardiac injury usually results from either gunshot wounds or stab wounds. Anatomically, one-third of the heart is situated towards the right of the midline and two-thirds lie to the left. This anatomical location creates a descending order of injury incidence with the right ventricle (RV, 40-43%), left ventricle (34-40%), right atrium (18-24%), and left atrium (3-5%) [4]. All these injuries can be fatal injuries, and they carry an overall mortality rate of 47% [4]. A computed tomography (CT) scan is the gold standard for confirming cardiac trauma in patients who have a positive history, abnormal electrocardiogram (ECG), or abnormal troponin levels [5,6]. Recent advances in the field of portable ultrasound, including focused-assessment-with-sonography-for-trauma (FAST) scans, have made it possible to perform a simple, quick, and non-invasive assessment of cardiac trauma [7].

## Case presentation

Patient 1

**Figure 1:**
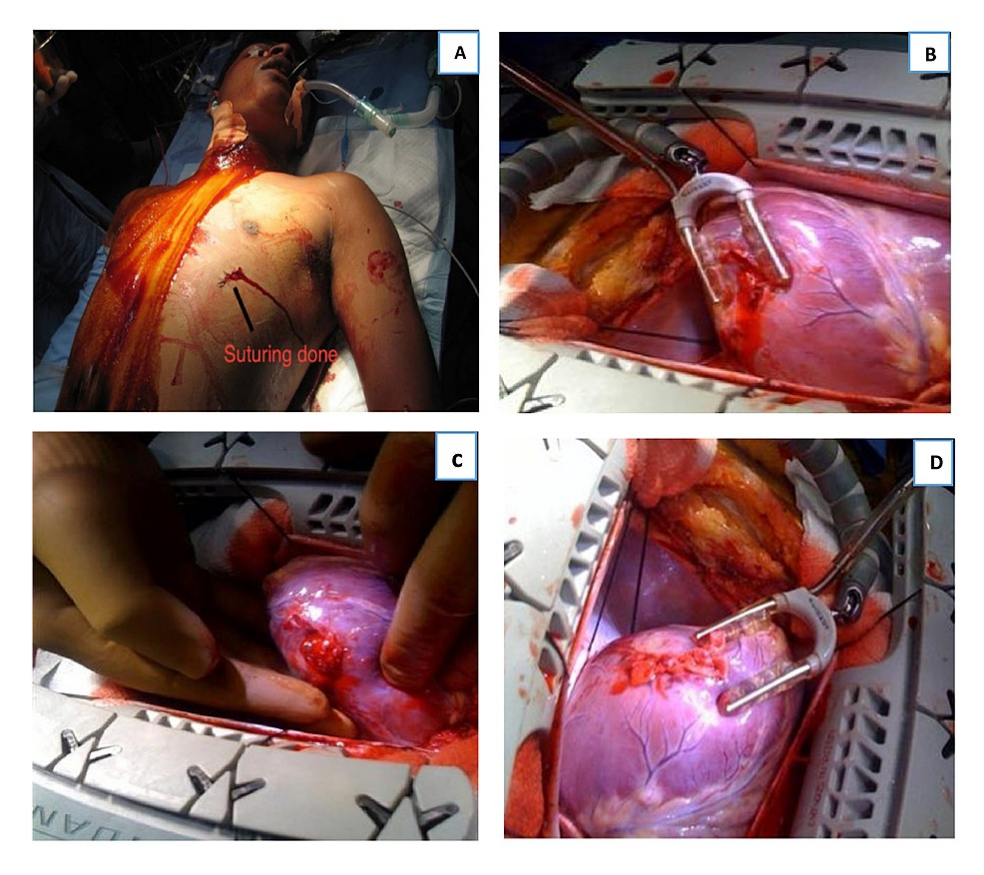
Stab wound on the anterior aspect of the left side of the chest (A). Intraoperative picture showing tear in the lateral wall of the left ventricle between the LAD and OM territory (B, C) and after the repair of the tear with Teflon felt pledgetted sutures (D). LAD: left anterior descending, OM: obtuse marginal.

A 14-year-old boy was brought to our hospital four hours after sustaining a stab wound on the left side of his chest. He had been seen immediately after the accident by an outside physician where his superficial wound was sutured (Figure 1A). Due to the continuous oozing of his injury site, his parents decided to bring him to our tertiary care center. Upon examination, the patient was agitated and pale, and hence Advanced Trauma Life Support (ATLS) protocol resuscitation was initiated. FAST examination revealed moderate pericardial effusion. Two large intravenous lines were immediately placed, and labs were ordered. The patient was taken to the operating room as hemodynamics were maintained. However, after anesthesia induction, the patient had a cardiac arrest. Cardiopulmonary resuscitation was initiated and a flash sternotomy was done, which revealed a tense pericardium. Sinusal cardiac rhythm was restored once pericardiotomy was done and collected blood drained. His heart showed good contractility and his BP normalized as well. The transesophageal echocardiography (TEE) did not reveal any ventricular septal defects (VSDs) or valve injuries. Intraoperative exploration identified a 4-cm partial tear in the lateral wall of the left ventricle which was located between the left anterior descending (LAD) and the obtuse marginal (OM) territory. Two sponges were placed beneath the heart and deep pericardial sutures to expose the lateral wall. The tear was repaired using 3-0 prolene sutures (on the beating heart) and reinforced with Teflon felt pledges on the clean-cut wound using an octopus stabilizer (Figure 1B-1D). The left ventricular wall surrounding the tear was not friable, which facilitated the suturing and the bleeding ceased. A left pleurotomy was then performed and 1 liter of blood was drained. Additional injury to the lingula of the left lung was identified and treated with local resection. After hemostasis, drains were inserted and standard closure was done. His postoperative recovery was uneventful; there was no neurological deficit, and the patient was discharged on day 7.

Patient 2

**Figure 2:**
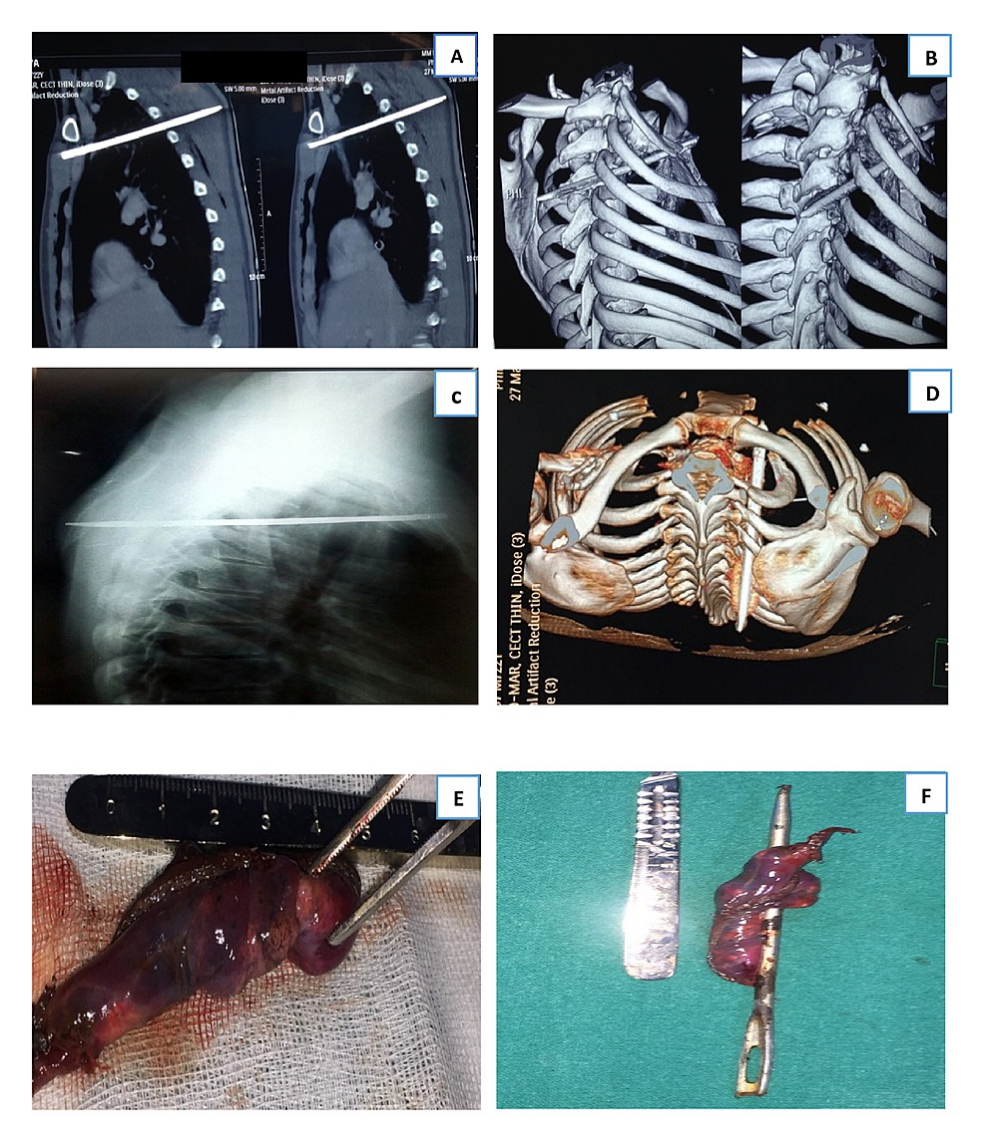
CT scan of the chest (A, B, D) showing spindle piercing the right anterior chest wall into the apical segment of the right lung and through the posterior chest wall. X-ray chest showing the same spindle passing through and through the right lung apex. The spindle after being removed from the body by surgical exploration (E, F).

A 20-year-old patient was referred to our center following an impaling injury with a spindle that had penetrated the anterior chest wall just inferior to the right clavicle. In addition to moderate pericardial effusion and no vascular injury (Figure 2A, 2B, 2D), the scan revealed the spindle traversing the right upper lobe of the lung. Due to resource constraints, a sternotomy approach was performed so as to assess any other associated cardiac injuries. Upon exploration, a small area of localized injury to the right lung apex was detected. A wedge resection of the right lung apical segment was performed, and the spindle was completely removed (Figure 2E, 2F) without any further damage to surrounding structures, and drainage of the associated pericardial effusion was performed. There was no associated injury to cardiac or other vascular structures. The patient made an uneventful recovery and was discharged on postoperative day 5.

Patient 3

**Figure 3:**
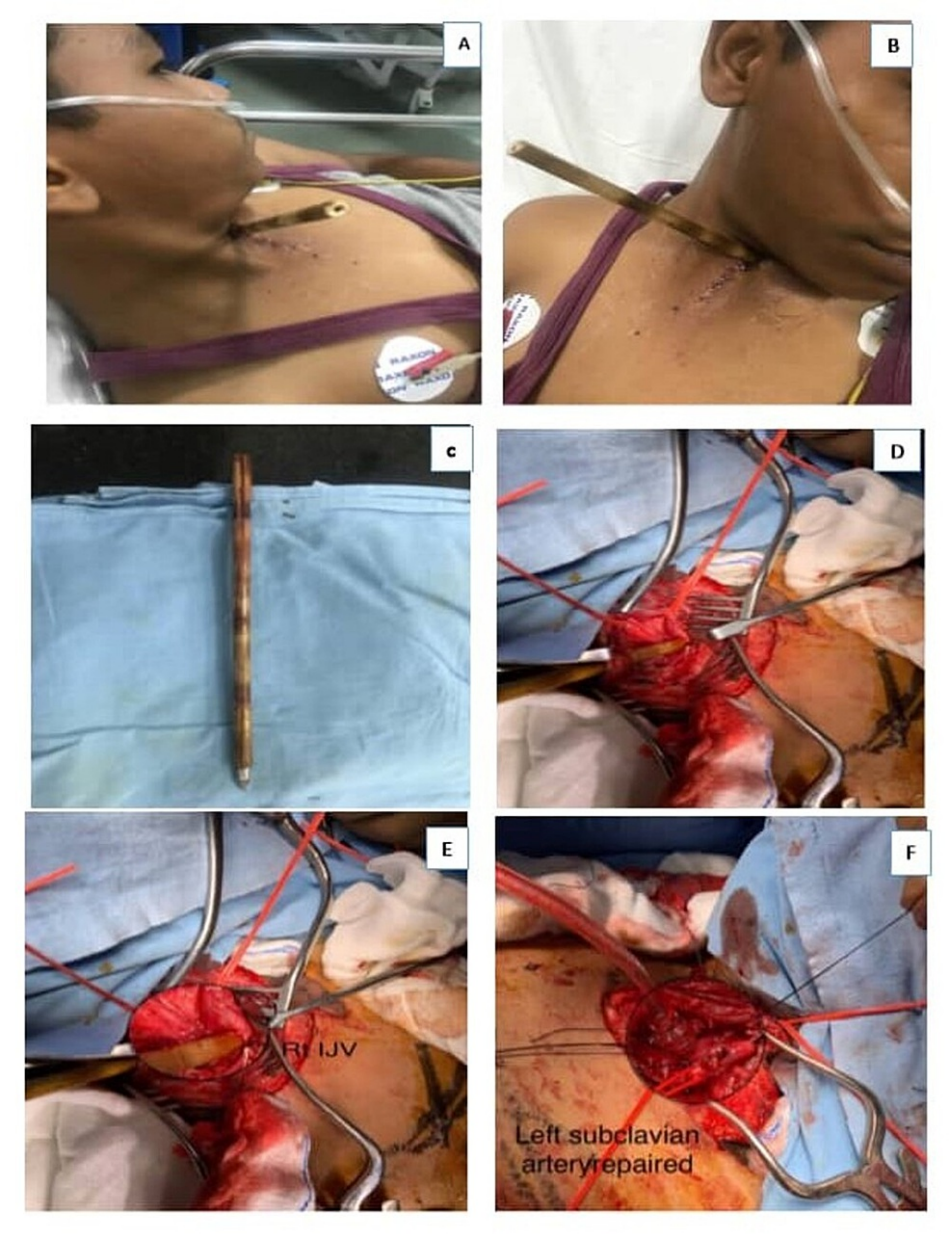
Arrow injury to the left side of the neck (A, B). Arrow after being removed from the neck (C), rent in the left subclavian artery (D, E) and being repaired (F).

A 14-year-old archery student was referred from an outside facility to our hospital following impalement by an arrow to the left side of the neck (Figure 3A, 3B). To facilitate transportation for advanced medical care, the arrow’s edges were carefully cut. An emergent CT scan showed that the arrow had penetrated the left side of the neck, transfixing the left internal jugular vein (IJV), which had thrombosed along with a puncture of the left lung apex. There was no associated tracheobronchial injury or pneumomediastinum. The patient was taken for immediate surgical exploration of the neck. In view of suspicion of injury to the left subclavian artery, a left anterolateral thoracotomy was performed in addition. There was continuous bleeding from the apical region consequent to the subclavian artery injury, which was repaired with 5-0 prolene once the arrow was removed (Figure 3D-3F). The repair required the clavicle bone to be cut, which was later fixed by the orthopedic surgeon. The patient made an uneventful postoperative recovery and was discharged home on day 10.

Patient 4

**Figure 4:**
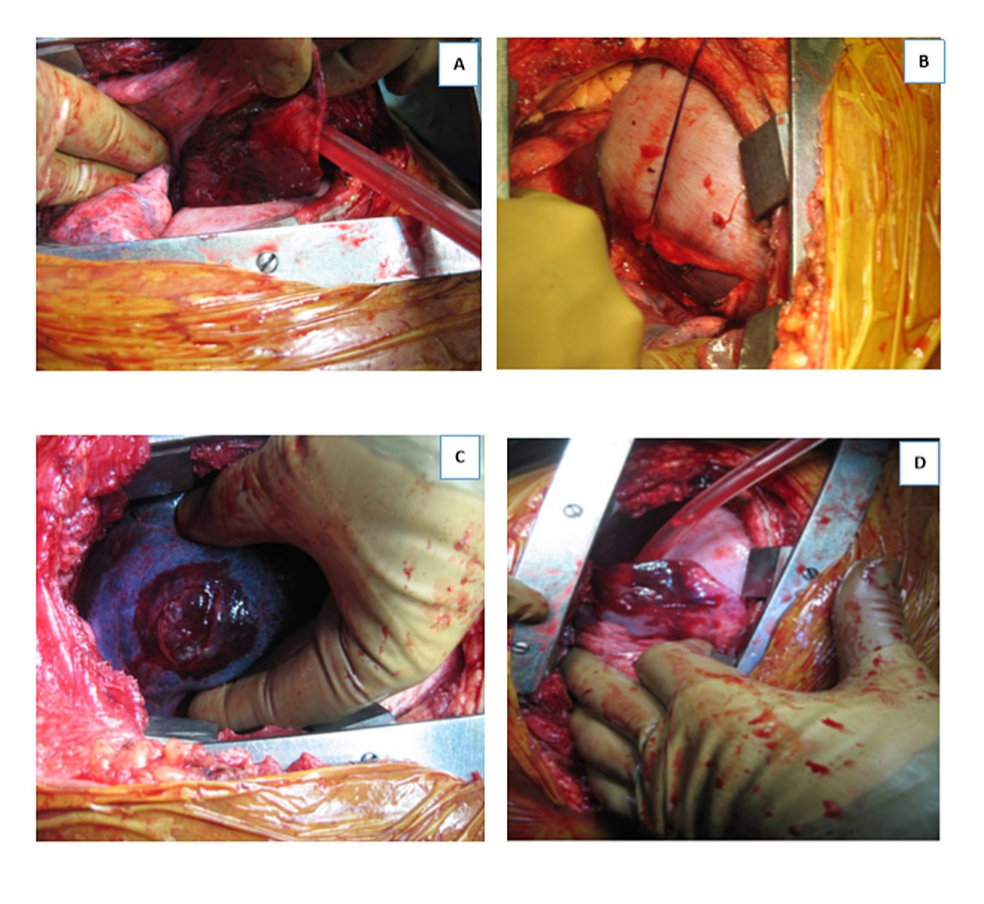
Intraoperative picture showing injury to the left lung (A, B, D) and diaphragmatic injury (B). Entry site of the stab wound on the left lower anterior chest (E).

A 50-year-old man presented to our hospital following a stab wound to the left lower chest. He was hemodynamically stable and FAST revealed no pericardial effusion. After initial resuscitation, left thoracotomy exploration was done in view of penetrating chest trauma, and he was found to have a diaphragmatic injury and a left lower lung laceration (Figure 4). The diaphragm was repaired with interrupted sutures using No.1 PDS, whereas the lung laceration was repaired with a purse-string suture using 5/0 Prolene. Exploratory laparotomy showed hemoperitoneum, which found a lesion of the omentum. No hepatic injury was identified.

## Discussion

Review of literature

Blunt Cardiac Trauma

Pathophysiology: Direct impact on the chest wall imparts a kinetic force to the patient, leading to compression of the heart between the spine and the sternum. This compression can lead to disruption of both the pump action of the heart and the momentum of the blood. Cardiac injury can also indirectly occur from blunt abdominal trauma, which could lead to the upper displacement of the viscera and indirect trauma to the heart [8].

Symptoms of cardiac injury after blunt cardiac trauma include hypotension, anemia from secondary bleeding, coronary vasoconstriction, and myocardial necrosis [8]. Blunt cardiac injury is often part of polytrauma. Other extracardiac symptoms associated with blunt cardiac trauma include rib fractures, sternal fractures, pneumothorax, hemothorax, organ injuries, and pulmonary contusion [9,10]. Up to 30% of blunt trauma patients have cardiac injuries, the most common cause being motor vehicle accidents [11]. Blunt cardiac injuries can be classified into various categories, which include: (1) injury with free wall rupture, (2) injury with septal rupture, (3) injury with coronary artery rupture, (4) injury with cardiac failure, (5) injury with complex arrhythmias, and (6) injury with minor ECG or cardiac enzyme abnormalities.

Manifestations, Diagnosis, and Treatment of Blunt Cardiac Trauma

Cardiac contusions: A cardiac contusion is the most common result of blunt cardiac injury. Although the diagnosis is quite difficult, it has distinct pathologic and biochemical abnormalities [8]. Various pathological findings from cardiac contusions include subepicardial and subendocardial petechiae, bruises, hematomas, lacerations, and full-thickness myocardial damage and necrosis [8,9,12]. From a histological perspective, leukocyte infiltration, edema, intramyocardial hemorrhage, and disruption of the myocardial fibers may occur in cardiac contusions [8,12]. A contusion can be complicated by the development of dysrhythmias, both atrial and ventricular, which may further compromise hemodynamics [8]. Atrial fibrillation was reported on the initial ECG in 4% of patients with nonpenetrating chest trauma [13]. The diagnosis of cardiac contusions can be made with a patient history positive for trauma combined with sinus tachycardia, ventricular extrasystoles, atrial arrhythmias, conduction abnormalities, or nonspecific ST-T changes observed on ECG [14]. However, it should be noted that there is no hallmark diagnostic pattern for cardiac contusions. Elevated creatine phosphokinase myocardial band (CPK-MB) isoenzymes, assay for cardiac troponin-T (cTT), and gated radionuclide angiography may be used to support the diagnosis [5,6,14]. Transesophageal echocardiography has proven to be quite sensitive for detecting myocardial contusions as well [14]. The treatment for a myocardial contusion is quite like that of a myocardial infarction without the need for thrombolytic therapy and anticoagulants [14]. Patients should be observed for at least two-to-three days to monitor for arrhythmias and congestive heart failure.

Cardiac rupture: Cardiac rupture is another manifestation of blunt cardiac trauma and can occur in many forms, such as rupture of the free wall, heart valves, papillary muscles, or chordae [14]. The rupture can occur acutely from the direct force of the injury, or the rupture may be delayed for up to two weeks due to myocardial necrosis [14]. Complete wall rupture is often fatal in most cases. A pseudoaneurysm can develop in which the rupture is sealed by a hematoma and the pericardium [10,15]. The most common presenting symptom of cardiac rupture is cardiac tamponade [14]. Suspicion of cardiac rupture should be high in these patients for an expert surgical consultation to be provided quickly. Diagnosis can be made through transthoracic echocardiography (TTE). In certain challenging cases, an MRI or TEE study can be helpful in getting a proper assessment of the heart [15]. In a patient who is unstable hemodynamically, the appropriate treatment is as per advanced cardiac life support (ACLS) guidelines. If the patient is hemodynamically stable, surgical correction of the rupture is done to prevent hemodynamic deterioration [16].

Pericardial injury: Although rare, injury to the pericardium after blunt cardiac trauma can be in various forms: pericardial lacerations, rupture, or pneumopericardium [16]. The pericardium is usually affected in any post-trauma setting and may occur several weeks after the traumatic incident [16]. In certain severe situations, frank hemopericardium and tamponade might occur. Furthermore, there is a risk of cardiac herniation due to laceration of the pericardium, which can either occur in the thoracic or abdominal cavity. Most commonly, the pericardial tears occur parallel to the left phrenic nerve (64%), along with the diaphragm (18%), and parallel to the right phrenic nerve (18%) [11]. In the case of herniation, the heart experiences impaired filling, becomes entrapped, and at times leads to compression of the coronary arteries [14,17]. The pericardial injury does not give rise to a hallmark set of symptoms. However, unexplained fluctuations in blood pressure and heart rate after a lateral deceleration injury should be cause for high suspicion, as the chances of developing constrictive pericarditis and pericardial rupture can remain for days to weeks [16]. Identification of pericardial injury can also be made through echocardiography as well as CT scans [16]. The treatment for pericardial injury involves total pericardiectomy. Through thoracotomy, the diagnosis can be confirmed, and the strangulation can be removed.

Valvular injuries: The last manifestation of blunt cardiac trauma is quite uncommon: valvular injuries. Because the mechanism consists of a sudden increase in intravascular or intracardiac pressure while the valve is closed, injury to specific valves of the heart depends on the contraction of the heart at the time of impact [11,16]. The aortic valve (the most injured) and pulmonary valves are most susceptible to injury in the early diastole phase, as empty ventricles provide little support and cushion for the impact on the heart [11,16]. Similarly, in early systole, the atrioventricular valves are highly prone to damage due to abrupt increases in intraventricular pressure, stretching the subvalvular apparatus [11,16,18]. As pressure is higher on the left side of the heart, the valves present on the left side are more susceptible to injuries [8]. It should be noted that mitral valve lesions occur because of rupturing of papillary muscles or chordae [18,19]. Rupture of the tricuspid valve during a MVA is the result of abrupt deceleration coupled with an increase in right-side cardiac pressures (Valsalva maneuver and thorax compression).

**Figure 5:**
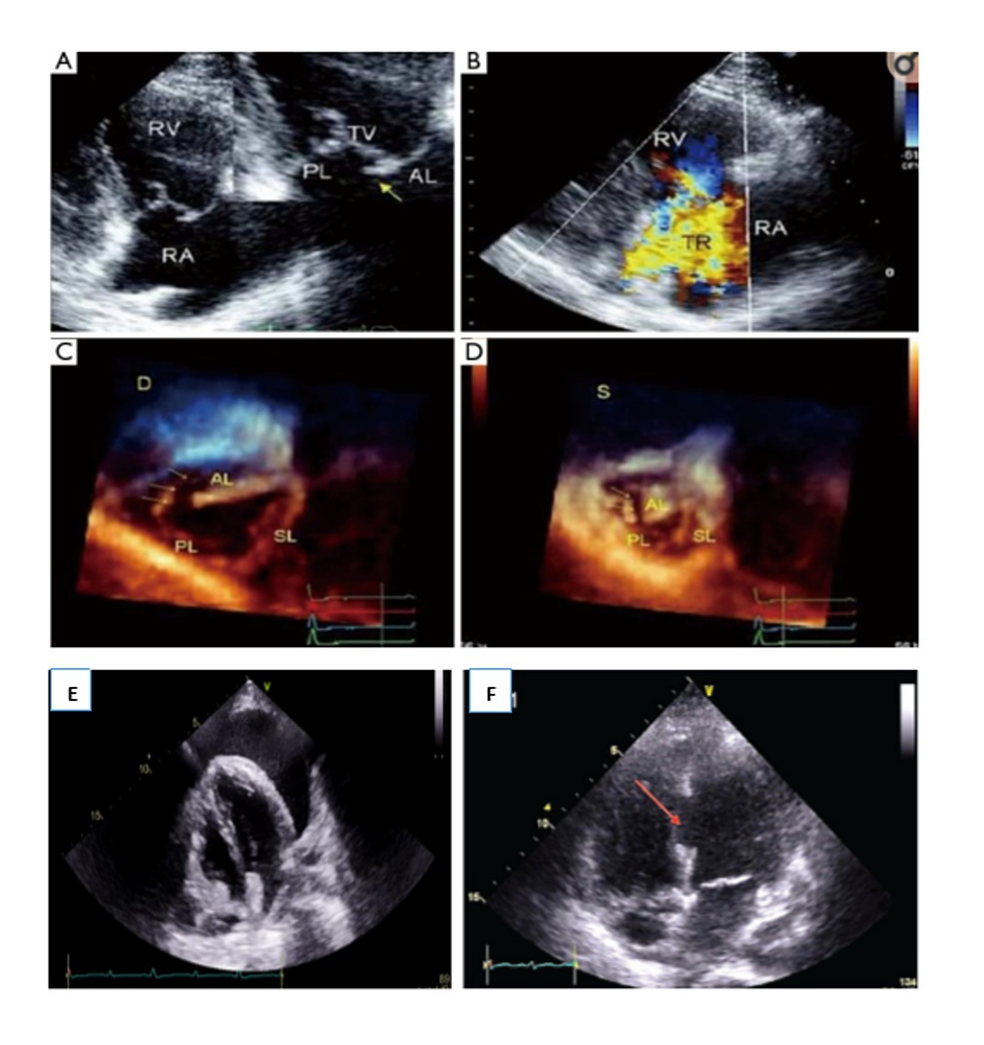
Transthoracic echocardiogram showing tricuspid valve injury (A, C, D), severe TR (B), pericardial effusion (E), and ventricular septal defect-red arrow (F).

Clinical manifestations include the presence of a heart murmur or the onset of congestive heart failure. Valvular injury can present with cardiogenic shock, but it may also remain asymptomatic for many days before giving rise to a severe clinical presentation [11]. Diagnosis can be easily done with echocardiography and CT imaging [10]. The presence of hypotension, ECG abnormalities, or markedly elevated troponin values should prompt further investigation with echocardiography in addition to expert consultation [10]. Severe valvular injuries require surgical intervention. Real-time 3D echocardiography helps to determine the timing of surgery in asymptomatic traumatic tricuspid rupture (Figure 5) [20,21]. The combination of echocardiography and magnetic resonance imaging provides information on volumetric data and contractility of the RV during follow-up. While ruptured chordae and annular tears can be repaired, papillary muscle rupture, however, may require valve replacement [11,18]. In selected cases, close follow-up can be sufficient as the severity of the injury may regress.

Ventricular septum injuries: Interventricular septal rupture resulting from blunt chest trauma is infrequent [22]. Although the exact mechanism remains unknown, some theorize that the rupture is due to the sudden compression of the heart during late diastole when the cardiac ventricles are full and the atrioventricular valves are closed, while others speculate that there is a microvascular disruption following the myocardial injury that ultimately leads to myocardial infarction of the septum [22,23]. Traumatic interventricular septum defects can be acutely symptomatic, with severe hemodynamic instability. However, in some cases, the onset can be more gradual when the rupture does not occur immediately after the trauma and manifests up to several years later [22,23]. Cardiac auscultation can find a holosystolic murmur along the left sternal border.

Transthoracic echocardiography remains the study of choice (Figure 5). In most cases, the interventricular septum is ruptured at its muscular portion [23,24]. However, traumatic membranous septal rupture following blunt chest trauma has also been reported [25]. Treatment varies based on the severity of the clinical presentation and the defect’s size. Large ventricular septum defects (>1 cm) and patients with hemodynamic instability require urgent surgical repair due to the increased risk of congestive heart failure (Figure 1) [23,26]. In moderate-sized defects, however, medical treatment is attempted first and the surgery is delayed by two to three weeks once the fibrous ring starts forming around the lesion, which facilitates suturing [23,24,26]. In other cases, where the defect is small, closure can occur spontaneously [23].

Cowley and Shaddy showed that postoperative and post-traumatic VSDs can be closed using Amplatzer devices with good outcomes [19,27]. In cases where surgical repair under cardiopulmonary bypass is not feasible, transcatheter treatment may be used [27].

Coronary artery injuries: They represent less than 2% of blunt chest trauma. Most commonly, there is a dissection and thrombosis resulting from intimal tears [11]. Other types of lesions include plaque rupture and epicardial hematoma [28]. It may, however, take days to weeks before the symptoms and signs occur. Coronary artery injury causes an acute myocardial infarction and presents with ST-segment elevation in the ECG territory of the affected artery [11]. Troponin is a sensitive marker and may be elevated [28]. Coronary artery injuries can also lead to tamponade in the case of artery transaction or pericardial rupture [11]. The most affected artery is the left anterior descending artery (76%), followed by the right coronary artery (12%) and the circumflex (6%) [29]. When suspected, coronary artery injury is evaluated by urgent cardiac catheterization and coronary angiography. In undiagnosed cases, complications that may arise include the development of persistent angina pectoris and the development of coronary artery aneurysm [17]. The management of such lesions varies and depends upon the mechanism, the comorbidities of the patient, and the degree of hemodynamic stability. It includes coronary artery bypass, patch angioplasty, and percutaneous coronary stenting. [11]. Hemodynamically stable patients with minimal injuries can be managed conservatively with anticoagulants and observation [29].

Commotio cordis: Commotio cordis is defined as sudden cardiac death resulting from a blunt and seemingly innocent blow to the chest in the absence of structural damage to the ribs, sternum, and heart and without the underlying cardiovascular disease [30]. It most commonly occurs in children, adolescents, and young adults in the context of participating in recreational and competitive sports such as baseball and football [30]. It is triggered by a sharp blow to the precordial area that occurs during the electrically vulnerable phase of repolarization (just before the T-wave peak), thus altering the cardiac electrical stability, which results in ventricular fibrillation or asystole [11,30]. The majority of commotio cordis patients do not survive. Factors contributing to the improvement of chances of survival include rapid recognition of the seriousness of the condition and initiation of CPR by bystanders with the use of automated external defibrillators (AEDs) [11,30,31]. In addition to raising awareness about this life-threatening arrhythmia that can be caused by a modest-seeming blow to the chest, it is important to improve the design of protective sports equipment [32]. Furthermore, given the life-saving role of AEDs, it would be recommended to have their availability in settings where commotio cordis is likely to occur [30].

**Figure 6:**
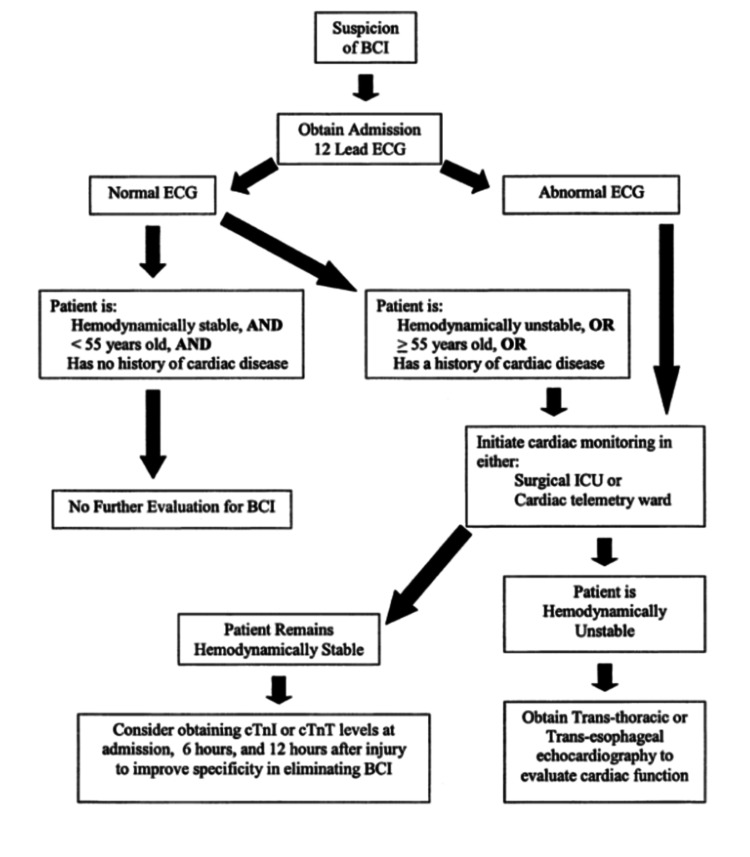
Algorithm for management of blunt cardiac injury

Initially, patients suspected of having a blunt cardiac injury should be evaluated with an ECG. If a patient has an abnormal ECG reading but remains hemodynamically stable, cardiac troponin I (cTnI) and cardiac troponin T (cTnT) levels should be drawn. If a patient is hemodynamically unstable, echocardiography should be performed to evaluate cardiac function (Figure 6).

Penetrating cardiac trauma

Pathophysiology

They are commonly caused by stab or gunshot wounds, or more rarely by accidental impalement [11]. Projectile injuries that penetrate the heart can be categorized into (1) contusion of the myocardium, (2) laceration and puncture of the chambers, (3) disruption/rupture of valves and leaflets, (4) disruption/perforation of the septum, and (5) injury to the coronary vessels [33]. Penetrating injuries have been found to be more fatal than blunt trauma. There is a risk of patients developing hemorrhage if no precautions are taken to prevent the wound from freely draining into the mediastinum or pleural space [33]. Another important cause of penetrating cardiac injuries is iatrogenic. Percutaneous coronary intervention, VSD repair, atrial myxoma resection, and even radiofrequency catheter ablation can cause cardiac injury, which commonly presents with dissection and hematoma formation [34,35].

Diagnosis

If the patient is stable (SBP > 90 mmHg), then the patient can be taken for X-ray and echocardiography imaging for further evaluation. Patients with an SBP <90 mmHg should be directly taken to the operating room for surgical exploration. In most cases, physical examination, chest and abdominal roentgenography, and echocardiography are sufficient to establish the extent of the injury [14]. Since the use of echocardiography for diagnosing penetrating wounds, the time required for the sound diagnosis of cardiac injury has decreased significantly [36]. The survival rate, as well as the neurological outcomes of the survivors, have also seen a positive transformation, and their quality of life has improved [36]. Furthermore, this diagnostic approach eliminates the need for diagnostic pericardiocentesis and subxiphoid pericardial window (SPW) procedures. In the case of projectile injuries, measuring central venous pressure (CVP), chest X-ray (CXR), CT, EKG, and echocardiography are the usual diagnostic procedures adopted [33].

Treatment

**Figure 7:**
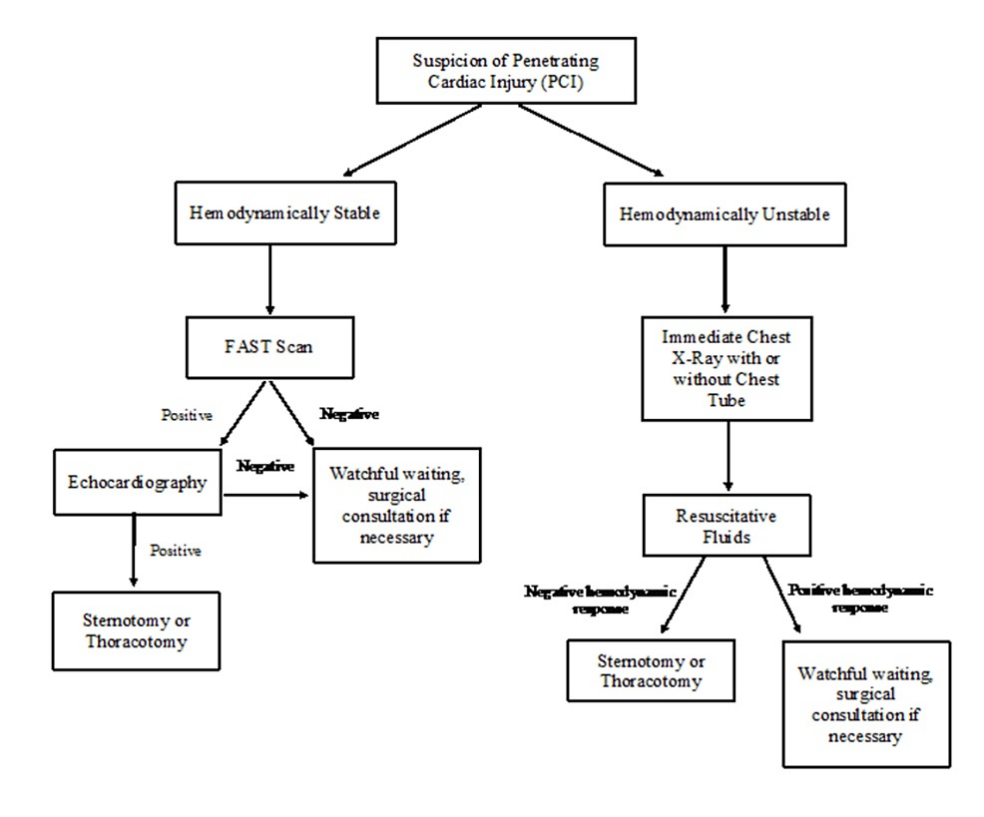
Treatment algorithm for management of penetrating cardiac injury.

The etiology of cardiac trauma is of critical importance. Small pericardial and myocardial wounds with tamponade can easily be treated with pericardiocentesis. However, larger wounds of the pericardium and myocardium resulting from bullet injury should be managed by immediate thoracotomy and echocardiography [33]. Once the injury has been identified, repair methods should be implemented with several basic aims: to relieve tamponade, stop the bleeding, and restore circulating volume [37]. If the myocardium is damaged, it should be treated with interrupted sutures. Damaged small coronary arteries can be handled and treated with simple ligation [38]. Figure 7 summarizes a rapid decision-making algorithm based on hemodynamic stability. Penetrating cardiac injuries require operative intervention with cardiac exposure best accomplished by either a left anterior-lateral thoracotomy or a median sternotomy [39]. A left anterolateral thoracotomy (emergency department, ED) provides quick cardiac exposure, which may be accomplished as a resuscitative thoracotomy [39]. An incision should be made in the fifth intercostal space from the sternum to the midaxillary line. Exposure provides immediate pericardial decompression and allows for aortic cross-clamping [39]. If there is a myocardial injury, there are a variety of ways to perform the cardiac repair. Finger occlusion and stapling with a skin stapler can be used for some temporary control [39]. Definitive repair is achieved with suturing, often with non-absorbable suture material. Pledgets may be used depending upon the requirements.

With the advancement of endovascular treatments, one of the new devices, REBOA (resuscitative endovascular balloon occlusion of the aorta), is a safe and effective option for emergency thoracotomy in patients with hemorrhagic shock secondary to noncompressible torso hemorrhage.

For patients with penetrating cardiac injuries who are hemodynamically stable, a FAST scan should be performed [40]. If hemodynamically unstable, a CXR with a chest tube may be considered in addition to fluids and possible surgical intervention. Figure 7 shows the algorithm for the management of a patient with a penetrating cardiac injury (Figure 7) [40].

A ten-year retrospective study of a regional trauma center indicated that with the introduction of immediate 2D echocardiography in the ER, the time required for the diagnosis of penetrating cardiac injury had been reduced and both the survival rate and neurologic outcome of survivors had seen great improvement [36]. In another retrospective study stretching over 22 years at a level I trauma center, 192 patients with penetrating cardiac injury, cardiac tamponade was the most common clinical presentation. The most common clinical intervention was tube thoracostomy, and the overall mortality rate was 25% [41]. The procedure of pericardiocentesis for diagnosis has declined over the years, with surgeons relying more on ultrasound of the pericardium for both normotensive and slightly hypotensive patients [41].

In a study conducted in South Africa over a period of three years, it was observed that 35 out of the 70 individuals who reached the hospital alive died, including all four gunshot wound victims [42]. Factors that aided in survival were isolated injury, the presence of cardiac tamponade (univariate and multivariate analysis), RV injury, single cardiac chamber injury, and the absence of pleural breach (univariate analysis alone) [42]. This study was followed by another retrospective cross-sectional study of penetrating cardiac trauma from 2000 to 2015, which reported a mortality rate of up to 20% [43]. They found the most common penetrating trauma causes were gunshot wounds and stab wounds. While this study had a small sample size (n=10), common intra-operative findings included pericardial tamponade and hemothorax. Median sternotomy was used to treat this penetrating cardiac trauma with 8 out of 10 patients surviving.

In their retrospective cross-sectional study conducted from January 1999 to October 2009 on patients with penetrating cardiac injuries, Isaza-Restrepo et al. indicated the prevalence of stab wounds. Based on the characteristics of the trauma, patients, and survival rate, there was most likely a high pre-hospitalization mortality rate [44]. Patients exhibiting cardiac tamponade showed a higher rate of mortality. Surgical management was satisfactorily using the pericardial window as the diagnostic method and sternotomy as the surgical approach.

In their study from 1983 to 1988, Brathwaite et al. identified 32 patients with blunt cardiac trauma. Out of these 32 patients, 26 died, indicating a mortality rate of 81.3% [45]. Despite rapid prehospital transportation, a high index of suspicion, and quick and efficient surgical intervention, survival can be dismal in blunt cardiac trauma. In a meta-analysis of blunt cardiac trauma, a total of 2,210 patients were included in the 25 prospective studies and 16 retrospective studies, for a total of 2,471 patients. In this meta-analysis, abnormal ECG and abnormal CPK-MB were shown to be associated with the development of clinically significant cardiac complications, whereas radionuclide scanning was not useful in the evaluation of patients with blunt cardiac trauma [46].

In their study, Decavèle et al. analyzed the associations between pre-hospital conditions, cTnI concentration, and mortality in intensive care unit (ICU)-admitted patients after trauma. They found that cTnI release was an independent predictor of ICU mortality with a concentration-response relationship (OR 4.90 [2.19-11.16] and 14.83 [4.68-49.90] for intermediate and high concentrations, respectively), which suggests that it can be used for stratification of early death in the ICU. In addition, their study showed a strong association between thoracic trauma severity and cTnI release [47]. Similarly, a recent study in Iran concluded that elevated cardiac troponin I and T levels provide excellent prognostic information regarding mortality in patients with multiple trauma, independent of age, hemodynamic variables, and Glasgow Coma Scale (GCS) score [48]. In contrast, Edouard et al. concluded that cTnI could not be used as a prognostic factor in trauma patients. Based on the results of their study, the sensitivity, specificity, and positive and negative predictive values of troponin I for the diagnosis of myocardial contusion were 63%, 98%, 40%, and 98%, respectively [49]. Furthermore, Kespaik et al. reviewed the prognostic value of elevated high-sensitivity cardiac troponin T (hs-cTnT) and found that it was correlated with a poorer outcome compared to patients with normal levels [50].

In their review of ED thoracotomy for patients suffering traumatic cardiac arrest, Nevins et al. demonstrated improved survival for penetrating vs blunt trauma (OR 2.10; p = 0.0028); stab vs gunshot (OR 5.45; p < 0.0001); signs of life (SOL) on admission vs no SOL (OR 5.36; p < 0.0001); and SOL in the field vs no SOL (OR 19.39; p < 0.0001). Equivalence of survival was demonstrated between cardiothoracic versus non-cardiothoracic injuries (OR 1.038; p = 1.000). Survival was worse for USA vs non-USA cohorts (OR 1.59; p = 0.0012) [51].

Nicole et al. in their study of hemodynamically stable patients with a hemopericardium (after X-ray, ECG, and ultrasound were undertaken), confirmed at the SPW, that no active bleeding was randomized. The primary outcome measure was survival to discharge from the hospital. Secondary outcomes were complications and a postoperative hospital stay. Fifty-one of the 55 patients (93%) randomized to sternotomy had either no cardiac injury or a tangential injury, whereas only four patients had penetrating wounds to the endocardium and all had sealed. There was one death postoperatively among the 111 patients (0.9%) in the sternotomy group. The mean ICU stay for a sternotomy was 2.04 days (range, 0-25 days) compared with 0.25 days (range, 0-2) for the drainage (P < 0.001). The estimated mean difference highlighted a stay of 1.8 days shorter in the ICU for the drainage group (95% CI: 0.8-2.7). The total hospital stay was significantly shorter in the SPW group (P < 0.001; 95% CI: 1.4-3.3 [52].

In 2007, Kokotsakis et al. published a case series where they discussed the use of intravenous adenosine to induce temporary asystole to facilitate the repair of penetrating cardiac wounds by offering a motionless surgical field. They reported positive outcomes [53]. Similarly, in 2002, Gill et al. supported that this method was better than induced ventricular fibrillation because it renders the heart more easily manipulated, particularly in lateral wall injuries [54]. Use of adenosine has also been reported in a case of cardiac penetrating injury with a foreign body retained in situ. In the absence of a heart-lung machine, asystole was chemically induced by the intravenous injection of adenosine and the nail was successfully removed [55].

A retrospective study by Karmy-Jones et al. in the tertiary care trauma center of the University of Alberta from 1992 to 1994 of all penetrating cardiac injuries found that three of eight patients survived; and that all patients who arrived with no vital signs died. The authors concluded that pre-hospital time is an important prognostic factor, as is the status of the vital signs on arrival. As such, patients who arrive with detectable life signs should be aggressively resuscitated. The authors also recommended that not all patients should benefit from emergency room thoracotomy (ERT) and that decisions should be based on presentation, available resources, and the team’s experience [56]. Similarly, Embrey reports that ERT is an effective treatment for penetrating chest injuries in patients who present with shock or require CPR for less than 15 minutes. However, it is a difficult procedure that yields favorable outcomes only when performed by experienced operators [11].

In 2011, an autopsy study was performed to identify the incidence and patterns of thoracic aortic injuries (TTA) in a series of blunt traumatic deaths and describe their associated injuries. The study showed that over 1/3rd of blunt trauma deceased victims had a thoracic aortic injury. A motor vehicle collision was the most common mechanism of injury (50%), followed by a pedestrian struck (37%). Patients with a traumatic thoracic aortic injury were more likely to die at the scene (80% vs. 63%, p=0.002). Additionally, they had a significant increased risk of sustaining associated injuries, including cardiac injuries (44% vs. 25%, p=0.001); hemothorax (86% vs. 56%, p<0.001), rib fractures (86% vs. 72%, p=0.006, and intra-abdominal injuries (74% vs.49%,p<0.001) [57].

In 2010, Kaptein et al. used the National Trauma Data Bank (NTDB) to study the epidemiology of pediatric traumatic cardiac injuries. They found that out of 626 patients, 69% sustained blunt trauma, which was associated with a contusion in most cases, and 35% had penetrating injuries. In-hospital mortality was 40%, and gunshot wounds had the highest mortality rate. Aside from the severity of the injury, the authors state that the excessive mortality rate could also be explained by delays in diagnosis. In terms of associated injuries, the study showed that rib fractures and lung contusions are the most common. Based on their findings, the authors suggested that the presence of pulmonary contusion, particularly when associated with rib fractures, should raise suspicion of cardiac injury in the pediatric population [58].

A retrospective analysis spread over a period of five years studied the reports of 380 motor vehicle occupants’ fatalities resulting from car crashes. It found that 21.1% presented with cardiac injuries, which was the most common cause of death or a major contributing factor to the fatal outcomes when present. In their series, the authors found that the pericardium and the ventricles were the most frequently affected cardiac areas, and the most common forms of injury were lacerations and contusions. Additionally, the study showed that sternal and left-sided rib fractures are predictors of an associated cardiac injury in victims of MVAs [59].

Turan et al retrospectively analyzed autopsy reports of fatalities caused by blunt trauma in a period of 3 years (2001-2003). Of a total of 1597 deaths attributable to blunt trauma, cardiac injuries were found in 190 cases (11.9%) and they were the cause of death or a major contributing factor in 45.2% of the cases. Vehicle accidents were the most common mechanism of injury (56%), followed by falls (38%). In terms of cardiac injuries patterns, pericardial tearing was the most frequently identified lesion (25%) and was associated with great vessel injuries in (28.8%) of the cases and with atrial and/or ventricular rupture in 38.5% of the cases. This could be explained by the vulnerability of the pericardium to acceleration and deceleration forces. The authors found no correlation between the injury pattern and the survival time. However, they hypothesized that patients with atrial and/or ventricular contusions are more likely to die later because of late arrhythmic complications [12].

In 2010, Kaptein et al used the National Trauma Data Bank (NTDB) to study the epidemiology of pediatric traumatic cardiac injuries. They found that out of 626 patients, 69% sustained blunt trauma which was associated with a contusion in most cases, and 35% had penetrating injuries. In-hospital mortality was 40% and gunshot wounds had the highest mortality rate. Aside from the severity of the injury, the authors state that the excessive mortality rate could also be explained by delays in diagnosis. In terms of associated injuries, the study showed that rib fractures and lung contusions are the most common. Based on their findings, the authors suggested that the presence of pulmonary contusion, particularly when associated with rib fractures, should raise suspicion for cardiac injury in the pediatric population [58].

A retrospective analysis spread over a period of 5 years studied the reports of 380 motor vehicle occupants’ fatalities resulting from car crashes. It found that 21.1% presented with cardiac injuries which were the most common cause of death or a major contributing factor for the fatal outcomes when present. In their series, the authors found that the pericardium and the ventricles were the most frequently affected cardiac area and the most common form of injury were lacerations and contusions. Additionally, the study showed that sternal and left-sided rib fractures are a predictor of an associated cardiac injury in victims of MVAs [59].

Turan et al. retrospectively analyzed autopsy reports of fatalities caused by blunt trauma over a period of three years (2001-2003). Of a total of 1597 deaths attributable to blunt trauma, cardiac injuries were found in 190 cases (11.9%), and they were the cause of death or a major contributing factor in 45.2% of the cases. The most common mechanism of injury was a vehicle accident (56%), followed by falls (38%). In terms of cardiac injury patterns, pericardial tearing was the most frequently identified lesion (25%) and was associated with great vessel injuries in 28.8% of the cases and with atrial and/or ventricular rupture in 38.5% of the cases. This could be explained by the vulnerability of the pericardium to acceleration and deceleration forces. The authors found no correlation between the injury pattern and the survival time. However, they hypothesized that patients with atrial and/or ventricular contusions are more likely to die later because of late arrhythmic complications [12].

Lung and Tracheobronchial Injury

Operative management of lung injury is uncommonly needed; delay in patients requiring treatment increases mortality and morbidity. Lung injuries following blunt trauma are less common and most often due to displaced rib fractures, which may cause significant tearing of the pulmonary parenchyma. In contrast, penetrating lung injuries result in lung laceration and hemopneumothorax and require knowledge of multiple approaches and operative interventions. The goal of treatment is to control hemorrhage and air leaks. Digital pressure on the pulmonary hilum can be used to rapidly control bleeding until the source is delineated. Surgical options to treat lung injury include pneumonorraphy, wedge resection or lobectomy, tractotomy, pneumonectomy, and evacuation of the hematoma [60].

Diaphragmatic Injury

Diaphragmatic injuries are usually associated with other thoracic and abdominal organ injuries. Although diaphragmatic injury can be obvious (e.g., herniation of abdominal contents on a chest radiograph), the injury may be subtle, and imaging studies can be nondiagnostic. A high index of suspicion needs to be maintained because delayed diagnosis is associated with an increased risk of herniation and strangulation of abdominal organs, which can be life-threatening. For patients in whom the diagnosis is uncertain, diagnostic laparoscopy, thoracoscopy, or open surgical exploration may be needed to establish the diagnosis. When identified, diaphragm injury is repaired with open surgical or minimally invasive techniques, the choice and timing of which depends upon the presence of associated injuries and the overall condition of the patient [61].

## Conclusions

Cardiothoracic trauma is an important cause of morbidity and mortality. Injuries to the heart and lungs abound in literature and date back as far as can be seen. The great wars and subsequent conflicts with high-velocity missiles drew attention to the devastation that can be caused to organs in the chest, among other injuries. MASH units (Mobile Army Surgical Hospitals) were conceived and developed by Dr. DeBakey and other surgical consultants in the first years of World War II and greatly improved survival rates. Improvised transportation and the addition of helicopters for transporting the wounded have contributed immensely to survival and mitigating the damage.

A variety of injuries such as penetrating injuries, firearm injuries, blunt injuries, and motor vehicle accidents contribute to the spectrum of cardiothoracic trauma seen in emergency rooms (ERs) across the globe. While the heart has captured the imagination of poets as the "seat of the soul" and the lungs as the life-giving breath, it suffices to say injuries to these organs can be lethal. A high index of suspicion followed by prompt referral to expert centers ensures optimum care and improved survival.
